# "The Hospital" Nursing Mirror

**Published:** 1899-12-09

**Authors:** 


"The Hospital" Nursing Mirror.

Being the Nursing Section of "The Hospital."

[Contributions for this Section of "The Hospital" should be addressed to the Editor, The Hospital, 28 & 29, Southampton Street, Strand, London, W.C., and should have the word "Nursing" plainly written in left-hand top corner of the envelope.]

### Notes on News from the Nursing World.

### THE QUEEN AND THE AMERICAN NURSES.

Monday was a memorable day for the American nurses who are still in London awaiting the departure of the "Maine" on Monday, the 18th inst. They confess that they will never forget their visit to Windsor. While they were delighted with the castle and warmly appreciated the hospitality shown them in the Queen's name, their interview with the Sovereign herself after luncheon was the great event which they will always look back upon with pride. Miss Hibbard, Miss Ludekins, Miss Manby, Miss Macpherson, and Miss MacVean were each personally presented by Princess Christian to her Majesty, with whom was Princess Henry of Battenberg. Her reception of them was characteristic. "I am very pleased," said the Queen, who seemed deeply interested, "to see you, and I wish to say how sweet it is of you, and how much I appreciate your kindness in coming over to take care of my men." The graceful tribute of the Queen to her guests and the motherly allusion to the sick and wounded troops as "my men," have impressed not only Miss Hibbard and her colleagues, but all classes of the community. Subsequently, the nurses drove to Cumberland Lodge and took tea with the Princess Christian. Many were the questions asked them in the evening at the Hotel Cecil—where they appeared in the dresses of white linen worn at Windsor, with stringless caps of the same material, and a brassard representing the Geneva Cross—respecting their experiences at Court. But they did not spend the evening in talking; on the contrary, they were foremost in their efforts to secure customers for the artistic programme prepared by the Society of American Women in London, and they collected a very substantial sum in boxes. Finally, they took part, along with the officers and doctors of the American hospital ship, in the tableau representing the "Maine," and received an enthusiastic greeting from the crowded and representative company. The Queen has given to the ship a splendid Union Jack, which the Duke of Connaught will present in Her Majesty's name on Saturday, the 16th.

### THE PRINCESS OF WALES'S WAR FUND.

In reply to our invitation last week we have received a very large number of letters from our readers expressing their desire that we should open a list of subscriptions to the Princess of Wales's hospital ship, and we have, therefore, much pleasure in complying with their wish. Full particulars will be announced next week.

### THE AMERICAN AMBASSADOR ON MISS FLORENCE NIGHTINGALE.

Among the guests at the banquet of the American Society in London were the surgeons and nurses who constitute the staff of the "Maine." The nurses were in uniform. In responding to the toast of "Our Ambassador" Mr. Choate referred to the letter written by Miss Florence Nightingale, in her retirement, enclosing a contribution to the fund for providing Tam o' Shanters for the soldiers in South Africa, and added: "When I saw the nurses in uniform enter the room I could not but think that Miss Nightingale's example had been an inspiration all around the world for this great and good work of women for the relief of the sick, the wounded, and the dying under whatever flag and on whichever side." No portion of his Excellency's speech was more warmly applauded by the company than this.

### THE NURSES OF THE "PORTLAND HOSPITAL."

The "Portland Hospital," which leaves on Saturday for South Africa, originated with Dr. Stokes, and Mrs. Bagot, wife of Captain Bagot, M.P., the Duke of Portland giving £5,000 to start it. The nurses chosen by the Sub-Committee, presided over by the Duchess of Bedford, who meet daily at the house of General Eaton, 43, Upper Grosvenor Street, are Nurses Davies, Russell, Pretty, and Cox-Davies. At a meeting of the committee on Monday it was announced that a retired naval officer was sending 300 tins of Bovril, and that through the Countess Bective and other friends cases of sterilised milk, prepared soups, jellies, and other delicacies for the sick wards in the hospital were being provided for shipment by the "Majestic." Through Mr. Anthony Bowlby, the senior civil surgeon, who accompanies the ambulance, a quantity of hospital shirts, cushions, pillows, arm-rests, and articles of linen have also been promised.

### THE INDIAN ARMY NURSING SERVICE.

Miss A. M. Harris has been gazetted a nursing sister in the Indian army nursing service. The ladies of the I.A.N.S. are, a correspondent states, somewhat indignant that none of their number have been selected to accompany the Indian contingent to South Africa. The reason given by the Government is that they cannot be spared from India, but the sisters argue that if the troops, medical officers, and hospital establishments can be spared surely two or three sisters could be spared also. The premature closure of the trooping season has a special interest for the sisters of the I.A.N.S. who have completed five years' service in India and are entitled to a year's leave to England this cold season, as they would in the ordinary way be granted passages in the troopships. They will now only receive Rs. 600 for their passage, or at best be granted a passage in an "outside" line of steamers.

### AN EMBRYO ARMY NURSE.

The church of All Saints, Portsmouth, stands a little back from the Commercial Road, and the open space around it is a favourite playground for the ragged children who swarm in that neighbourhood. On one side there are much-spiked iron gates. A correspondent who was passing the other day saw some children lift a little boy of four years old on to one of the bars. The fat baby naturally fell, and in falling a spike tore his leg. At the sight of the blood he howled lustily. Our correspondent says, "I was just about to sacrifice my pocket-handkerchief to stop the bleeding when a small, dirty scrap of humanity of about eight years old came running towards us, and shouting as she ran, 'I'll do 'un! I'll do 'un!' Pulling up her ragged frock, she extracted from a pocket in her petticoat a long piece of rag. 'We've been learning first aid' (at the Board school) 'and I always keeps a bandage in me pocket, and looks out for accidents. I've done up a lot!' Kneeling against the gate, she tenderly took the little foot in her lap and quickly and deftly bandaged the leg. The child was not much hurt, but frightened at the sight of the blood, and operator and patient went off hand-in-hand made quite happy by a few pence to buy 'sweeties.' Who can say, after this, that the 'first aid' instruction given in the Board schools is wasted on the children?"

### THE QUALIFICATIONS FOR COLONIAL NURSING.

In an interesting article, which appears elsewhere in our pages, a contributor, whose nursing experience in the Far East is considerable, intimates that the idea at home is that any nurse, however indifferent here, will be a shining light in the colonies. But, thanks to the Colonial Nursing Association, this idea, whatever may have been the case in the past, is rapidly fading away. Under its auspices none but properly qualified nurses, whose credentials are absolutely unimpeachable, are dispatched to tend the sick and suffering in Greater Britain. Thus, a nurse who sailed for Japan on Monday, and one who is being sent out to Singapore, at the instance of the Association, have both been trained in admirable schools and thoroughly well-equipped to practice their profession in any part of the world.

### ARE HOME CHARITIES IN DANGER?

In the report read last Thursday at the annual meeting of the Brighton, Hove, and Preston Nursing Association it was stated that the subscription list fell considerably short of the sum required. The Rev. Arthur Cocks, who seconded the adoption of the report, said it was most extraordinary to him that a charity like that should be in debt.

"It was not often one found a charity about which everybody spoke well, but during the three or four years the Jubilee nurses had been working in Brighton the only complaint he had heard urged against them was one of his own; and that was that as soon as one got to know and appreciate a nurse she was removed, but he was bound to say she was replaced by one equally charming and skilful. Indeed, the association seemed to have an inexhaustible supply of exceedingly charming and skilful nurses, and the thing which struck one most about them was the absence of their professionalism in the home of the patient—they were not working simply as nurses but as good women, who had the interest of the particular cases they were attending thoroughly at heart. That was the reason why they were so popular and so well received in the districts in which they ministered."

Mr. Cocks went on to enter an earnest plea against support hitherto given to local charities being diverted into other channels, and hinted that, owing to the war, home charities are in very serious danger of diminishing. He is not the first who has suggested such a catastrophe, but we confidently hope that the people who are subscribing so generously to the various War Relief Funds will not "rob Peter to pay Paul." It would be a disgrace to the nation if, while it contributed freely to help the sick and wounded soldiers it left the sick and injured at home to die.

### BELFAST NURSES' HOME.

Mr. H. O. Arnold Forster, M.P., was one of the speakers at the annual meeting of the Belfast Nurses' Home last week. He said he had had the happiness of being under the care of nurses himself, that he happened at one time in his life to do a certain amount of work in a general hospital, and that some of the best and brightest of his friends had given the whole of their lives to that great profession. He congratulated Belfast in being in the front rank in the matter of the standard of nurses, and observed that "in that respect their circumstances were much happier than was the case in other parts of Ireland." From the report of the home we learn that the staff of sisters and nurses is 105; and that as regards the work of the last year, in addition to nursing in the Royal Belfast Hospital, the nurses have attended 483 private cases.

### INDOOR UNIFORM AT PUBLIC ENTERTAINMENTS.

With reference to our comments on the question of indoor uniform at public entertainments, and the visit of a sister and eight probationers in uniform to a concert at Manchester, our correspondent states that since she wrote to us the hospital matron has given six nurses tickets to a dance, "to which they went, by her arrangement, in the said indoor uniform." Our correspondent continues, "I should like to ask if you consider that some should be allowed the privilege and others not? I am correct in saying that the nurses knew before going that the Guardians saw no reason why they should not please themselves, as the tickets were being given; also that the hospital matron had given permission for the uniform to be worn at dramatic entertainments for which tickets had been sent, &c. I have attended annual dances for nurses and staff for the past 15 years, but they have been held in our own hospitals (not private affairs), and I am quite of the opinion that it is not right to so use it. But I venture to think that there is a wide difference between an intellectual treat in music and dancing for pastime."

### NURSES WITH BLACK BAGS.

During the discussion at the meeting of the General Medical Council Mr. Brown said he did not object to the education of nurses, but he objected to uneducated women acting practically as doctors, or going beyond their duties as nurses. His reference was to the unqualified persons who, under present conditions, are allowed to act as midwives; and he mentioned a case in his own experience where he found, on being called in, "a nurse not with a supply of linen, but a black bag, which contained medicines and instruments." Black bags, of course, may be perfectly harmless, but black bags with medicines and instruments are not among the proper equipments of a trained nurse; but then we suppose that when Mr. Brown spoke of a nurse it was a midwife that he meant. Quite another thing.

### OUR CLOTHING DISTRIBUTION.

We have already received half-a-dozen parcels of clothing, for which we tender our heartiest thanks to the senders. Policy No. 1,081 forwards eighteen articles of well-chosen, beautifully-made clothing, amongst which are some charming baby socks; H. P. contributes two garments; N. C. Grist, three; Nurse Hillman, eight; Nurse Harris, four; and Miss N. Tallack, fifteen —in all fifty good and useful articles, including men's shirts, women's petticoats, and children's frocks. Nurse Mary Rolerson also sends a parcel, most of the contents being the work of her patients. We are anticipating the receipt of many more similar offerings to our hospital sick, and we therefore ask intending contributors if they will kindly send their parcels in as soon as possible. As Christmas Day falls on Monday, all gifts should be sent to The Hospital about the middle of the preceding week.

### ASKING TOO MUCH.

A fortnight ago Miss Fenelly, of Waterford, was appointed trained nurse to the Claremorris Union, at a salary of £70 a year. She has just addressed a letter to the Clerk of the Board of Guardians acknowledging her appointment, and stating that she must have a thorough understanding with the Guardians before she enters upon her duties. She says:

"I will not be satisfied with the medical officer's arrangement, because I have some experience of such. As a matter of fact, no trained nurse can work with an untrained one, and those 'old dames' who are generally favourites of the doctor and the board as well. I will not consent, under any circumstances, to be placed on a level with an untrained person, whether religious or lay. You say I will be head nurse, then why am I not in charge of the hospital? No untrained person or so-called nurse will boss me."

Miss Fenelly, after stipulating that, if she is on night duty, she must have comfortable apartments in the hospital, and that her attendant must be a person able to cook well and keep her apartments clean, continues:

"Let me know if my rooms are close to the hospital, and if I will have one entire ward to pass through during the night, or if I will be confined under the same roof. Why I am so particular about all these details is simply, I may tell now, because I don't intend to upset myself going so far, and perhaps have to leave it again, so that I want everything settled satisfactorily before going, in order that peace may reign supreme. Of course you will understand that I will not do anything in the form of servant's work."

It will hardly be a matter of surprise that, after the letter had been read to the chairman and 30 other Guardians, the Clerk was instructed to write to the young lady, informing her that the conditions and requirements laid down in her letter were wholly unacceptable.

### RECREATION FOR NURSES.

An excellent example has been set by Miss Norman, the lady superintendent of the Eastbourne Nursing Institution, who has just given an afternoon "At Home," followed by a dance in the evening, to which, in addition to her own social circle, her staff of nurses were allowed to invite their personal friends. Both functions proved most enjoyable, though, of course, the dance found greater favour among the younger people. The nurses in their specially devised uniforms of pale blue silk zephyr, and wearing their nursing caps and aprons, made a picturesque appearance among the non-professional guests.

### THE HAMPSHIRE NURSING ASSOCIATION.

It has very properly been decided, at the instance of the Countess of Selborne, to increase the salary of the superintendent of the Hampshire Nursing Association to the amount paid by other organisations. Not only is Hampshire the largest county association, with 37 districts belonging to it, but Miss Phelps, the superintendent, is indefatigable in the discharge of her duties. At the annual meeting it was also decided to supply each district with a time-table book to be kept by the nurses and submitted once a year to the secretary, the book to be open to the inspection of the nursing association. The object in view is to obviate individual inquiries as to the work of the nurses. It is a matter for regret that the association, owing to lack of sufficient resources, has had to keep some districts waiting many months for a nurse, and a matter for satisfaction that the candidates applying to the association for training are of the right sort.

### HALIFAX INFIRMARY AND HEBDENBRIDGE NURSING INSTITUTION.

At the annual meeting of the Hebdenbridge and District Nursing Institution the other day several speakers laid stress on the needs of the Halifax Infirmary. One of them observed that "its finances were in an awful condition, and that the committee were spending something like £1,200 a year in excess of their income." He added that it had been mooted at their meetings whether it would not be necessary to close one, if not two, of the wards. Another speaker said that, though the Hebdenbridge people sent a great many more cases to the infirmary than formerly, he did not think that they sent any more money. We agree with him that this is not an ideal state of things. The subscriptions should correspond in amount to the cases sent to the infirmary, especially as the latter is in such urgent need of funds. It is, however, pleasant to learn that, though the nurses at Hebdenbridge consider it a dull place, they have no inclination to go out to South Africa, but are quite content to look after poor sick people in Yorkshire.

### SHORT ITEMS.

It is satisfactory to learn that the work of the nurses in Armagh is appreciated by the public. At the annual meeting of the district nursing association, when all classes and churches were represented, the report, which was unanimously adopted, showed that 6,769 visits were paid by the two nurses in the year ending last September, and that the association has a balance to its credit of £178.—In order to pay for extensions to the nurses' home and laundry in connexion with the Bristol Royal Infirmary, the Governors have decided to sell out sufficient capital funds to realise about £8,000. The cost of the home itself was £7,700.—The first annual meeting of the Oakengates and District Nursing Association was held last month, when a satisfactory financial report was read, showing a balance in hand of £7. The nurse, whose work was much appreciated by both patients and doctors, had attended 104 cases, and paid 2,353 visits during the year. It was recommended that the patients should be encouraged to contribute.—On Saturday Sir Richard Thorne Thorne gave a lecture on the Prevention of Tuberculosis to the nurses of the Royal British Nurses' Association at 11, Chandos Street, Cavendish Square. Dr. Pavy took the chair. The subject was one which aroused the keenest interest, judging by the large assembly of nurses, who numbered nearly a hundred.—In answer to a telegram of congratulation to the Princess of Wales on her birthday, sent on behalf of the inmates of the British Home and Hospital for Incurables, Streatham, the secretary (Mr. R. G. Salmond) has received the following gracious reply: "Very much touched by my poor incurables' kind wishes and thoughts. Hope they will accept some pheasants.—Princess of Wales."

### Lectures to Surgical Nurses.

By H. A. LATIMER, M.D. (Dunelm), M.R.C.S. of Eng., L.S.A. of London, Consulting Surgeon, Swansea Hospital; Past President of the Swansea Medical Society; Lecturer and Examiner of the St. John Ambulance Association, &c.

### BILIARY AND URINARY CALCULI.

I SHALL devote this lecture to the subject of concretions in our bodies—calculi or stones, as they are generally called. There is a particular liability to the formation of these tormenting substances in the biliary and urinary organs, and, whilst they produce their most distressing effects here, they are also to be met with elsewhere—as in the nose and in the intestines. In the latter situation they may give rise to conditions of great gravity by blocking the canal and so leading to obstruction of the bowels, with all its secondary dangers of peritonitis; in such an event the operation of laparotomy will be called for, and you will have to nurse the patient as in other illnesses where the abdomen has been opened for the removal of any surgical disease in that region. So I will dismiss this part of my subject with the few remarks I have made on it, and will turn to the consideration of stones as they affect the neighbourhood of the liver and the kidneys.

When present in either of these parts they set up an agonising condition of pain by their attempts to escape through narrow passages which exist for the purpose of conveying bile or urine into the intestine, in the first case, and into the bladder in the second. For the liver and the kidneys are organs which exist for the purposes—among other functions—of secreting from the blood liquids which we term bile and urine; and when these liquids have been formed they are conveyed away through the bile ducts and ureters to their appropriate destinations. Now the key to understanding how stones form in these regions is to remember that the bile and urine are fluids containing in solution various bodies which are capable of crystallising into solid substances if, from various causes, the fluids in which they float in the dissolved state alter their character. You can go on dissolving a soluble substance in an appropriate fluid until the quantity put there has reached what is called the "point of saturation," and then you will find that what you add of the substance will lie undissolved at the bottom of the liquid. And you, doubtless, have noticed that if you alter the condition of the dissolving fluid by heat it will have the effect of dissolving some more of the substance, which will again reappear as a solid when the warm fluid is allowed to cool. Let me give you a very ordinary illustration of what I am saying. I take, for instance, a bottle and put in it a quantity of phosphate of soda, and then I fill the bottle with water. I observe that a good deal of the soda has been dissolved but that a portion of it still settles at the bottom of the bottle. I note that the water will only hold a certain amount of soda in solution; now I warm the water in the bottle and then shake its contents; the fluid becomes clear, and all the phosphate of soda has been dissolved. I now put the bottle aside and let it cool, and when cold I again find that phosphate of soda has deposited itself as a crystalline substance at the bottom of the bottle—in other words, it has come out of solution from a saturated liquid. This simple illustration is given merely for the purpose of showing that solids remain unseen in liquids under appropriate conditions; it does not explain why liquids, such as the bile and the urine, furnish us with gall-stones and urinary calculi. It shows only one side of the question, viz., that under certain circumstances these fluids are the sources of the concretions. I take it that you are not concerned with the circumstances which lead to the formation of calculi; it is sufficient for you that they exist as derangements of health of a well recognised character.

But before passing away from this part of my subject I must draw your attention to this fact in the subject of crystallisation: that if by any chance, in a fluid which is highly charged with a dissolved substance, a solid body appears, that solid body will attract to itself substances which have been hitherto dissolved in the fluid, and will, by such attraction and deposit, grow larger and larger. This is, no doubt, the way in which stones first form, and subsequently increase in size. Oftentimes a cold—a biliary or vesical catarrh—has resulted in the secretion of some mucus from the membranes lining the bile passages or the bladder. Very quickly this mucus attracts to itself crystalline substances hitherto dissolved in the bile or in the urine, and a stone is at once in process of formation. This solid body which acts as the attractive force is called a "nucleus," the word being derived from the Latin, and signifying a kernel; a further dictionary definition being given as "a central mass or point about which matter is formed."

It is interesting and curious to notice what bodies may act as nuclei for stones. I have seen a hair-pin removed from the bladder of a woman which, by becoming encrusted with phosphatic deposits, had served as the nucleus for a stone there; and I have myself removed a large phosphatic calculus from the bladder of a man in which the nucleus consisted of a portion of a red-rubber catheter which had been left behind in that cavity in the act of drawing off the urine. In the same way bile-stones have been found to have as a centre one or more minute worms.

When calculi have formed in the neighbourhood of the liver or kidneys, in attempting to escape therefrom they set up agonising pains, the peculiar features of which are that they are paroxysmal in character. A person suffering in this way is said to be attacked by biliary or renal colic. From time to time the patient is gripped with a series of pains, which increase in severity until a point of extreme agony is reached, when, in many cases, they suddenly cease. I think it is doubtful whether anyone who has not endured an attack of one of these varieties of colic can have an idea of the horribly painful and intensely depressing character of such an attack as I am speaking of. He may be able to form a conception of it, however, by observing anyone who is in the throes of such a paroxysm, for he will see the sufferer arrest his breathing as if afraid or unable to use the respiratory muscles, whilst the face will have a deathly aspect, and beads of perspiration will collect about the brow. Vomiting will also often occur. If the pain gathers in intensity from this point, fainting, or even death, may ensue from the mere paralysing effect of pain on the central nervous system, from whence it is reflected to the heart which it stops in its action. The pains run in definite directions, according to whether they are caused by bile or kidney stones—in the first case from the right side towards the stomach and one or other of the shoulders, in the second from the loins to the groins or the end of the water passage. They are undoubtedly caused by the irritation of nerves distributed in the walls of the bile-ducts or ureters. These channels, when invaded by the disturbing foreign bodies, contract upon them, and attempt to move them by energetic contractions of the muscles which they possess. It is a peculiarity of the muscles distributed in the walls of internal cavities that they act in successive waves, hence the paroxysmal nature of pains in these cases. Such pains cease either because the stones return to the part from whence they came or escape into the larger cavities of the bowel or bladder, when they cease to annoy: or they set up a fresh train of symptoms of their presence.

### Nursing American Soldiers.

### A VISIT AT NIGHT TO THE PRESIDIO HOSPITAL, SAN FRANCISCO.

By an English Nurse.

It was eleven p.m. when I arrived at the Presidio, armed with the necessary "permit" from Colonel Girard. My object was to see the inner workings of the hospital where "Uncle Sam's" brave but sick "boys in blue" were being nursed back to health and strength—or gently assisted into the shadowy land of the great hereafter. Outside the soldiers were settling in their tents or little sloping-roofed houses for the night.

The hospital looked big and awesome by moonlight. In the rear, opening out of the square court, are ten wards; within them long rows of beds filled with soldiers, all of them bodily sick, many homesick for the homes and friends they might never see again. The busiest part of the night was over, the baths, cold packs, &c., had been given in the fever wards, and the wounds dressed and done with for the night in the surgical wards, excepting those requiring hourly attention. The nurses, whose night duty lasted from half-past seven p.m. to half-past seven a.m., were now able to enjoy a minute or two's rest, to sit down and peruse one of the popular magazines of the day. Not for long, however, as owing to the numerous beds there were many calls to attend to.

I went first into one of the surgical wards. On the door were notices that no one could enter without a permit, and that the door was not to be slammed. My pass being exhibited, I was ushered in quietly by a kind-faced nurse, clad in the pretty blue linen uniform with white cuffs, collar, and apron—a nurse so like our own that the scene might have been one in England.

The light was screened, and it was not at first easy to distinguish the beds; but my eyes soon got accustomed to the dimness, and the firelight from the hospitable stove came to my assistance. The nurse motioned to me to warm myself thereby, which I gladly did. That was the "convalescent ward," she told me, but many of the poor fellows looked as if they had still a hard fight back to health to encounter. The nurse was soon called away. I watched her as she struck a match to select a drug from the case in the centre of the room, administered it, then calling to a male assistant with the red-cross badge, gave him charge while she was absent showing me round.

The surgery consists of a series of shiny white rooms, having a very antiseptic odour. One little speckless apartment is where the water is sterilised, and where, on shelf after shelf, medicines, bandages, and other necessaries abound galore. Then come the lavatories, and beyond them is the operating room, which is always interesting to the surgical nurse visitor. High and dazzling white walls surround the important operating room; in the centre stands the operating table awaiting its next victim. On glass tables are ranged the glittering "cruel to be kind" instruments. All was so spick and span that you might have eaten your dinner off the floor.

It was midnight, and the distant clock was striking the hour. The nurses left their work in the charge of their assistants, and went off to the nurses' building. There, in the kitchen, they enjoyed a brief little meal. A fine-looking woman dished them out an appetising mess of potatos and steak, and each brought her own cup for coffee. It was all swallowed post haste; occasional little scraps of conversation, mostly "shop," relieved the silence. I was handed on to a pretty little nurse to have a look at her surgical ward—a very busy ward it seemed, even in the "wee'sma' hours." The doctor was making his final visit for the night, taking genuine interest in the cases, listening patiently to details. If the shades of the dead soldiers of the war of '62 were looking on, they must have envied their companions in arms the care they were receiving in 1899. How different the skilful doctors, and dainty sweet nurses, to say nothing of the anæsthetics, unknown then, compared to the rough army doctor of the bygone day, assisted by the "Gamps" of the period.

The next visitor was a "cullud genelman," carrying in the coal hods, as black as his own complexion, which was relieved by his big red lips and shiny white teeth. Even he had a cheerful grin for us all on his ebony countenance as he attended to the two stoves.

Leaving that portion of the hospital, and traversing the long verandah—where it must be a treat for convalescents to promenade, I came to the typhoid ward, which contained forty men. Many of these were muttering in delirium, occasionally shouting; some having to be gently restrained, which was done in the firm, kind way one is accustomed to witness in our English hospitals. Others were tossing restlessly. There were the usual types of patients—the inevitable grumbler, the impatient, the grateful, and even the waggish. I had a little chat with some of them as I passed along. Most of them were grateful for their good care, but were impatient to get home to "Mother," or "my old woman an' the kids." But the very bravest had been better able to stand the hardships in the field than the prolonged tedious after care. I had a glimpse into a small ward where "two dying soldiers lay." Poor boys! Everything had been done for them that was possible, and the end was not far off. An assistant was sitting by them; the nurse gave a few directions to him. Then we returned to the large ward, where signs of dawn were appearing, a welcome sight to nurses and their charges.

Faces and hands were being washed, egg nogs and milk punch were being distributed to those who needed them. At seven o'clock a small waggon was rolled in containing a most appetizing assortment for breakfast for those who were far enough advanced to be allowed to partake of solid food. There were eggs, mush, steak, and coffee from the "mess room." The "specials" and "light" diets were looked after, and prepared by, the nurse in her ward kitchen. Then came the change of night nurses "off" duty and day nurses "on," and it was time for me to depart. So, with a few words of thanks to the courteous nurses, I left the "carbolicky" atmosphere for the lovely sea breeze outside, and passing near the little white tents dotting the landscape reflected favourably on the care "Uncle Sam" gives to his "boys in blue" when they need it, and also on his good taste in selecting such sweet "girls in blue" to alleviate their weariness and pain.

### Appointments.

Norfolk and Norwich Hospital.—Miss Dorothy Burroughes has been appointed Lady Superintendent. She was trained at Guy's five years; was three years superintendent of the Home for Girls, Mortimer Street; and on two occasions has been locum lady superintendent at the Norfolk and Norwich Hospital.

Grantham Hospital.—Miss Jeanie Chapman has been appointed Matron. She was trained at the South London Fever Hospital. She has since been three years staff nurse and four years sister at the Western Infirmary, Glasgow; and night superintendent at the Royal Infirmary, Bradford.

Chelsea Hospital for Women.—Miss Laura H. Ulph has been appointed Night Superintendent and Assistant Matron. She was trained at St. Bartholomew's Hospital. She has taken sister's duties at the Hospital for Sick Children, Great Ormond Street, during holiday time.

Margate Cottage Hospital.—Miss Rosa E. Bennion has been appointed Nurse-Matron. She was trained at St. Bartholomew's Hospital, and has since been on the private nursing staff.

### Colonial Nursing.

By a Nurse in the Far East.

There are some essential points of difference between nursing in an institution at home, where the nurse is a very small cog in well-oiled machinery, and in the hospital abroad, where in her position as sister she is sometimes expected to combine the qualifications of nurse and house surgeon; and from this point alone the necessity for fully-trained women will be made manifest. There are periods—especially during night duty—in which others than her own patients come under the sister's care, and to meet the emergencies constantly arising she must be prepared upon all points. Too much stress cannot be laid upon this feature, for the idea at home is that any individual, however indifferent there, will be a shining light abroad; and the mistake is a dire one. How can a nurse with mental training only cope with the exigencies of a critical surgical case, or the fever nurse with those of a maternity one? And, in addition to her other qualifications, she should be a lady, not necessarily of high birth, but at least a "nature's" gentlewoman, withan in nate sense of the fitness of things that will save her from wreckage when embarked upon the social round which claims more of her time and attention than is possible elsewhere.

First Impressions.

The ardent lover of her work will be distinctly disappointed with her first colonial experience. The days of a prompt obedience to her orders, of the doctor's regular visit, of the enjoyable clinical lecture, and of the absolute control of her ward are a thing of the past, for there are no trained probationers, no doctor but the one (in some places two), who is frequently called away for a few hours upon matters official, and no students to be lectured to. There are none but native boys and women, with a slight knowledge of English and a still slighter knowledge of nursing, to assist one; and as they both agree that the wishes of the patient (be he delirious or otherwise) and their own laziness are of primary importance, there is usually some loss of time between the giving of an order and its completion.

The Hours.

The eight-hour system in a tropical country is almost universally adopted, since the enervating climate tries one's powers at times to the uttermost; but while giving more freedom it has disadvantages, especially from the point of administration, for it is impossible to ensure the correct transmission of a thousand comparatively unimportant odds and ends from, say, the morning sister to the two who succeed her, and the result is often slight friction all round.

Then, too, the eight-hour system means a recurrent period of night duty at more or less irregular intervals, when any spare hand may be told off to replace the sister whose turn it is to become "owl," and in some cases one returns to find all one's pet theories and fads swept ruthlessly away. It is not always thus, however, and there are compensations, as may be seen later on.

The Absence of Asepticism.

Asepticism, so rigorously enforced at home, holds no sway here, and the days when, clothed in a sterilised over-all, the ward sister cleverly manipulated dressings and sponges by means of a pair of sterile tongs, occur to one's memory as a phantasy, a chimera, a dream. Given a bowl of 1 in 20 carbolic, a few marine sponges, unboiled instruments steeped in an antiseptic, and a more or less clean finger, our tropical surgeon is content, and proceeds to explore the abdominal cavity. Is there some occult virtue in the dressing used, or is it pure and simple idiosyncrasy that the patient occasionally recovers? It is difficult to say, for modern ideas do not accommodate themselves readily to lint soaked in carbolic oil upon which iodoform has been dusted as a substitute for aseptic or antiseptic gauzes in the case of operations for ovarian tumour or liver abscess.

No Theatres.

Theatres are a comparatively unknown quantity, and under the above circumstances might be deemed superfluous. We used frequently a small room set apart for cases of very bad enteric or delirium tremens, and in this ovariotomy has been performed with a successful result. These operations, however, are withheld if at all possible, and the patient is advised to go where climatic conditions cannot exercise a baleful influence, and she is wise if she accepts the advice so cordially tendered. In almost all colonies the nurse who is a good linguist will receive most appreciation from the patients, especially if her station happens to be at a coast port, where cosmopolitanism reigns supreme; and, as elsewhere, the many-sided nurse who plays a little, draws a little, and sings a little is always a favourite.

Opportunities for Study.

Her long off-duty hours admit of study, of outdoor exercise in all its varied forms, and as much social life as she cares to attempt in a land where the woman who has voluntarily forsworn home ties and associations in order to mitigate the sufferings of her fellow creatures is held in the very highest esteem, and is invited everywhere.

The Question of Climate.

Climate in the tropics is too much deprecated. There are seasons when moist or dry heat try everyone, but the curious part of it is that the individual who has definite and regular work seems to suffer less from its ill-effects than the one who, without employment, follows the bent of her feelings and does nothing. Who has not seen, while training, the delicate or anæmic member in her own household develop into a strong, capable nurse, simply under the influence of regular habits and meals of a plain but wholesome character? And abroad the careworn nurse who has passed through the curriculum in a London hospital is rejuvenated by her outdoor life and her absolute freedom from the wards in her off-duty time.

### Minor Appointments.

British Hospital, Algiers.—Miss Mary Thomson has been appointed Nurse. She was trained for three years at St. Saviour's Infirmary, East Dulwich, and for nearly two years has held the post of ward sister in the Poplar and Stepney Sick Asylum. Miss Maude Rodgers has also been appointed Nurse to this hospital. She was trained for three years at the New Infirmary, Birmingham, and since then has been staff nurse at the Heart Hospital, Soho Square, and ward sister at the Poplar and Stepney Sick Asylum.

Bury Union Workhouse.—Miss E. Ball has been appointed Superintendent Nurse. She was trained at the Workhouse Infirmary, Brownlow Hill, Liverpool, and has since been charge nurse at Edinburgh City Poor House, assistant superintendent at East End Mothers' Home, and night superintendent at Stockport Union Infirmary.

Tenbury Union Infirmary.—Miss J. F. Gore has been appointed Assistant to Matron. She was trained at Fort Pill Hospital, Chatham, and has since been nurse at Cockermouth Union Workhouse and at Fort Pill Hospital, Chatham.

Worcester Isolation Hospital.—Miss F. Hancock has been appointed Charge Nurse. She was trained at the City Hospital, Birmingham, and has since been charge nurse at Leyton Isolation Hospital.

Tewkesbury Hospital.—Miss L. Farley has been appointed Staff Nurse. She was trained at Rotherham Hospital, and has since been staff and theatre nurse.

### Royal British Nurses' Association.

A successful and most enjoyable conversazione was held by the Royal British Nurses' Association on Monday in the rooms of the Royal Institute of Painters in Water Colours, Piccadilly. Miss Thorold, Mr. Pickering Pick, Mrs. Coster, Mr. Langton, and Mr. Fardon received the guests, and some excellent entertainments were provided for their amusement. Songs, recitations, magical illusions, a humorous and musical selection by Mr. George Grossmith, and another by Mr. James Dunn, contributed to a varied programme, whilst the pleasing performances of the Æolian Ladies' Orchestra were much admired. Mr. George Grossmith was delightfully funny both in his songs and in his sketch of the country maiden leaving home for the first time. The scene was a first-class carriage, wherein the absent-minded damsel had settled herself and her innumerable parcels with a third-class ticket. Her private farewell, performed in public to the eight ladies who came to see her off; the hunt for her pocket in order to find her ticket; and the final wild scramble to change carriages evoked much laughter and applause. The musical mutoscope, exhibited for the first time, had all the charm of novelty; suitable music is supplied by the phonograph, whilst the appropriate action is seen in the moving picture. Thus one hears the dance music and sees the dancer at the same time. Dr. J. Blumfeld recited "The Absent-Minded Beggar," and a liberal contribution found its way into the "little tambourine" for the Reservist Fund. As Mr. Grossmith said in dedicating his encore song to his audience, "the sweet nurses' looked very "attractive" with their bright faces, in their fresh uniforms. Very nearly five hundred managed to be present at this pleasant annual gathering.

### British Home for Incurables.

On Monday there was an interesting function at the British Home and Hospital for Incurables at Streatham, the occasion being the unveiling of a new window in the chapel by the Lady Mayoress. The chapel, which has a parquet floor and is lighted by electricity, is a very pretty building and seats about 200 people, besides the gallery at the west end. There were groups of coloured chrysanthemums in pots within the sanctuary, and ferns and foliage at the altar step and around the pulpit. The Lady Mayoress arrived shortly after three o'clock, accompanied by Mr. Sheriff Bevan, Miss Ida Bevan, and Mrs. Treloar. After unveiling the east window, which is a beautiful work of art, the visitors repaired to one of the rooms, where a sale of work under the management of Lady Norman was opened by the Lady Mayoress, who was presented with a very handsome bouquet by Miss Carlyle, the oldest patient in the home. The corridors and rooms are very light and airy, and the papering of the former is very effective—a warm stone colour and artistic shade of blue, above a dark wooden rail and a dado of the stone colour. The building is not yet complete, and Sir Francis Norman, in thanking the Lady Mayoress for her presence, mentioned that a good-sized assembly room would shortly be completed and would be of great advantage to the institution.

### To Nurses.

**In order to increase and vary the interest in the *Mirror*, we invite contributions from any of our readers in the form of either an article, a paragraph, or information, and will pay a minimum of 5s. tor each contribution. All rejected manuscripts are returned in due course, and all payments fcr manuscripts used are made at the beginning of each quarter, i.e., January 1st, April 1st, July 1st, and October 1st.**

### Presentations.

Leeds Union Infirmary.—On Tuesday last week Miss Hopper, late matron of the Leeds Union Infirmary, who has just resigned in order to take the post of matron of the Bethnal Green New Infirmary, was presented by her nursing staff with a beautiful beaten silver tea pot and cream jug. Miss Hopper went to Leeds five years ago for the purpose of starting a system of trained nursing where before there had only been partially trained nursing, together with pauper assistance. During the past five years she has gathered round her and organised to a high degree of efficiency a staff of fifty nurses, and no one who has not undertaken the task knows the difficulties of building up such an edifice on the foundations of the old, and with some of its still remaining material. Miss Hopper has accomplished the work admirably, and has won the gratitude of the board and the admiration and affection of her entire staff of nurses. Mr. Councillor Lawson, late chairman of the Leeds Board of Guardians, made the presentation, and gave expression to the universal appreciation of Miss Hopper's services and the regret felt at her departure.

Irish Branch of Queen Victoria's Jubilee Institute.—Miss Dunn, general superintendent, Irish Branch Q.V.J.I., having resigned her post, which she has held since 1890, has been presented with a solid silver tea set as "A token of affectionate esteem and appreciation from Queen's nurses in Ireland past and present."—Mrs. Blackmore, assistant superintendent, Irish Branch Q.V.J.I., has been presented on her resignation with a handsome silver-mounted dressing-case, "With sincere good wishes from the Queen's nurses." Mrs. Blackmore has been district nursing since 1884, and joined the Q.V.J.I. in 1891.

Union Infirmary, Wakefield.—An interesting ceremony took place at the Union Infirmary, Wakefield, last week, when the nursing staff presented Miss Last, the superintendent, with a handsome pair of silver fish carvers bearing the inscription, "Presented to Miss A. Last by the nursing staff of the Union Infirmary, Wakefield, on the occasion of her marriage." Miss Last carries with her the good wishes of a great number of friends.

### Our Christmas Number.

With our Christmas number, to be issued next week, will be presented to our readers two full-page portraits of the Queen—the Queen Empress, and the Queen Mother—selected by Her Majesty, the first being a reproduction of the only photograph of the Queen taken in her robes of State. The letterpress will be entirely written by nurses, and will be entitled "On Wings of Love: An Angelic Visitation." As there will be a great demand for this number, orders for copies should be given without delay to the publisher.

### Novelties for Nurses.

### A FLANNEL CLEANSER.

Barrymore's "Special" Flannel Cleanser is the name of a useful preparation that seems destined to supply a long-felt want in the laundry world. We are all aware of the difficulty of getting flannels satisfactorily washed and the inevitable shrinkage that attends their more intimate acquaintance with the wash-tub. In the "flannel cleanser," however, we have a preparation that supersedes the use of soap, and renders the material delightfully clean and soft. It is in the form of a powder, and about a teaspoonful added to the water in which the flannel is to be washed will produce the desired result. It has also other virtues—it restores the brilliancy to faded silks and renovates coloured blouses and ribbons. It is also a refreshing adjunct to the bath, and can with advantage be used for cleansing the hair. It should be rubbed into the scalp with a little water sufficient to form a shampoo. It has the additional merit of softening the hardest water, which is an advantage that will be appreciated by dwellers in the metropolis. It is at least worth a trial, and we are bold enough to prophesy that it will result in the "cleanser" being added to the list of household requisites on its termination.

### A Few Hints for the Private Nursing of Typhoid.

BY A PRIVATE NURSE.

HAVE as little furniture in the room as possible, only strips of carpet that may be taken away and shaken or oil-cloth on the floor. Do all the tidying of the room yourself; never allow a servant to enter the room, and as few other people as possible. Wash the floor over daily with Jeyes' disinfectant. This should be done with a fairly dry cloth, not to make the boards too damp. Dust with a damp duster. Keep several bottles of carbolic lotion ready mixed (1 in 20). Calvert's No. 5 is suitable for this purpose; this is for use in the disinfection of stools and clothes, the full strength for the former, added to water for the latter. The strength to be used will be found written on the bottle. Never carry on a conversation in the room or near the door. Keep the light subdued. Have your patient on a narrow bed with a macintosh and draw-sheet over the under-sheet; if you have two small beds the same height, so much the better, as the patient can be easily drawn from one to the other, and consequently the mattress is able to be aired daily. Keep a blanket especially for rolling your patient in while sponging; a little vinegar added to the water when sponging will bring down the temperature more quickly than anything. When sponging, take off the patient's night-shirt, roll in the blanket, expose only one portion at the time, stroke with a gentle though decided touch towards the length of the body, dry with a dabbing movement, cover up, then proceed to the next part. Be sure and air the blanket and towels thoroughly after use. From the first rub the back and bony prominences with methylated spirits, and powder with zinc powder. A similar treatment very gently done will relieve the muscular tenderness so constant in the legs in this disease. Have the hair cut short; it is impossible to apply an ice bag properly without. Be very careful to keep the mouth clean, the teeth free from sordes. This may be best done by twisting a piece of wool round a forceps and dipping into a mixture of borax and glycerine, or equal parts of lemon juice and glycerine. Never dip the same piece of wool into the mixture twice, but pull it off with another forceps and put it into a piece of paper, wind another bit of wool round and repeat; burn the wool immediately after. Always keep a nail brush, or, better, two, on the washstand, also a second basin containing a solution of perchloride 1 in 2,000. Wash your hands frequently, and always after handling the patient bathe in the solution. Wash the thermometer after use, and put into a glass containing carbolic 1 in 20, with a piece of wool in the bottom and a paper cover on the top, with a hole in the centre just large enough for the thermometer to pass through.

Keep all nourishment outside the room, strain everything yourself, and give certain quantities at certain intervals; always record what and when they are given, and otherwise keep a faithful written report for the doctor's benefit of all incidents in connection with the patient. Keep at least three feeders in use, boil them once a day in soda water, keep the spouts scrupulously clean. Have a serviette always in readiness to place under the chin when giving nourishment. If possible obtain a cradle bed pan, keep it near the fire that it may be always warm and ready for use. Put the urinal into a thick woollen sock, empty the motion into a chamber kept in the lavatory, pour a strong solution of carbolic over it, cover with a cloth wrung out of carbolic lotion, and keep for the doctor's inspection, or for two or three hours before emptying down the drain. Measure the urine. If vomiting is troublesome try lime water with the milk. I often find this more successful than soda water; of course this is subject to the doctor's approval. If epistaxis is severe it may often be stopped by teazing out a piece of wool and lightly placing across the nostrils. Never ask your patient a question, always answer him truthfully. Don't laugh at his delusions, they are only too painfully real to him, but soothe him by tactful answers.

As long as there is life never give up hope of pulling him through; impress your hopeful views on his relations. Avoid any discussions with them as to the treatment ordered by the doctor or his opinions: politely tell them when they ask you that you consider it would be much more satisfactory for them to speak to him themselves.

### Private Nursing in Roumania.

BY AN OCCASIONAL CORRESPONDENT.

IN private nursing one meets with many different experiences, but the case that I am at present nursing is quite remarkable. To begin with, my patient is a Roumanian child, just a year and a half old, and born at eight months. Her father is a delicate man of 56 years, and her mother a Russian lady, delicate, but young. For twelve months, as is the custom of the country, she had a wet nurse, who was not at all satisfactory, and when she left the child was quite ill. When I arrived I found the little one perfectly white, without a particle of colour, unable to walk or speak, and evidently very delicate. She slept very badly at night, and only for about half an hour in the day. Whatever she wanted she had, and was generally mistress of the house. When she woke up in the morning, between four and five a.m., if she were not dressed at once she screamed to such an extent as to give one a headache. Then about going to bed, her mother would say, "Does baby want to go to bed?" and if she protested then she was allowed to stay up until eleven or twelve p.m. She had bonbons, sardines, herrings, potatoes, radishes, &c., to eat, and very rarely drank any milk. Her parents did not allow the nurse to let her cry, and in consequence this is the way the child was managed. When I arrived she would not look at me, and cried terribly whenever I touched her. Therefore she was immediately given back to her former nurse, a German, who was still in the house. It has taken three months for the child to get used to me, and now she is so loving she will not go to anyone else.

As Roumania is so hot I came with my patient into the Austrian Alps, and she has greatly improved. Baby's parents are followers of Kneipp and Lahmann, and she has no meat extract of any sort. I give her Frame Food and Mellin's Food. As she is so much better I shall soon be leaving, and I shall be very sorry, as I have grown quite attached to the child. She has such pretty ways, and can already walk alone and speak a little. She sleeps for three hours in the day, and from seven o'clock at night until six the next morning—quite a change to what she did three months ago. She is to have an English hospital nurse permanently, as she requires the greatest possible care. Her mother is tuberculous, and baby already shows a tendency towards that disease.

She has beautiful blue eyes, high cheek bones, slanting shoulders, and is perfectly blonde. Sometimes she looks like an angel, so that I fear she is not long for this world. Besides, she has such old-fashioned ways, and is far too intelligent for her age. She will never go to sleep unless I kiss her, and one day I was away for the whole day and found her awake upon my return. When I kissed her and put her into bed she was soon asleep. The nursing of my "angel patient," as I call her, has been one of my hardest cases, but one of the happiest, as I have daily witnessed the reward of perseverance and patience.

### Echoes from the Outside World.

### AN OPEN LETTER TO A HOSPITAL NURSE.

The last Cape mail brought a welcome letter from the sister of the ladies who have gone to Ladysmith. Writing from Durban, she says: "On Sunday I went with a friend to visit the 'Sumatra,' the P. and O. transport, to see the wounded regulars. We got up at half-past six, so as to make a good batch of scones to take with us, and also to pick sufficient flowers to be worth the offering. We got on board without hindrance soon after ten a.m. with our arms full, for we had brought as many magazines as possible in addition to the flowers and scones. It was hot, of course, below, as the vessel lay in dock, and we found the men in wooden cots made of planks fastened to uprights set in the floor and roof at even distances. Many of them had only been wounded on the Monday, and complained of being very sore from the railway journey, moving uneasily from side to side as if in much pain. The ambulance man in charge told us that, owing to the Durban Commandant's permit not having been received, the poor soldiers were kept standing or lying for two hours upon the wharf before the ship would receive them; and even when they got on board they were only given the same rations as they had been receiving at the front. Tea, without milk or sugar, with dry bread so badly made that it was like putty, morning and night; bully beef and potatoes for dinner; and never any change. Fancy such a diet for sick men who have nearly given their lives on our behalf!"

The writer continues: "There was hardly a man on board with a single wound—nearly all had two or three—yet most were bright and willing to talk, and all seemed glad of the flowers and scones. It is impossible to say too much of the pluck and endurance of the soldiers. They never uttered a word of complaint, and some were far too ill to have been put on board at all. We saw two of the officers to whom, you may remember, we gave tea at Gillitt's Station on their way to the front. Our recognition was mutual. One, belonging to the Irish Fusiliers, was lying in a deck chair with his wounded foot up. He said, 'Why, here you are! I never thought I should see you again!' He had been through that terrific march from Dundee to Ladysmith; then on the Monday he was with the detachment who were forced to lay down their arms because the mules had stampeded with their ammunition. He himself had been wounded, and he lay on the ground and heard the Boers discussing what they should do with the wounded. They decided that the prisoners were enough to manage, and that the wounded must be left. One commandeered Free Stater, an Englishman, offered to carry the officer down the hill, but the Boers told him that they would shoot him if he did, so that he had sorrowfully to depart. Later on a kind-hearted Boer took him in his arms, huge man as he is, and carried him to a form and laid him down, first giving him a coat to rest on instead of the damp ground. Thus he remained till daybreak; but during the night two of his wounded men crept painfully down the hill and joined him. There they all three lay till the ambulance found them next day, he told us. In the afternoon we drove down to the wharf again, and handed over to the ambulance man a couple of cooked fowls, some tins of milk, and packets of sugar, some cake and home-made bread, the result of a collection amongst our neighbours, and were so rejoiced to hear that someone had just sent 30 lb. of butter. We have all resolved that our heroes shall not suffer for lack of good food again. As long as it is necessary, some of the members of the Ladies' Patriotic League will meet the trains as they come down, and see that the poor fellows have the comforts they require."

The full list of casualties at Modder River, which was published on Saturday evening, though, of necessity, it brought with it sorrow to many a home, also to a certain extent relieved the apprehensions of the multitude. Lord Methuen's despatch had aroused fears that the total of the dead and wounded would prove to be terrific, partly because Lord Methuen said that the battle had been "one of the hardest and most trying fights in the annals of the British army," but more especially because a newspaper correspondent, telegraphing at the same time, stated that "the bloodiest battle of the century" had taken place. It was amazing how soon this suggestive phrase was taken up. It seemed to spread like wildfire; poor and rich alike appeared to have heard it, and, worst of all, in a short time it was firmly believed by most of those who helped to spread the report that they were quoting the words of the official telegram. Probably the meaning of Lord Methuen's words was exactly what he said, without any idea of breaking to us gently horrors to come. How could a battle be anything but "trying" when the enemy were entrenched on both sides of a river, the bridge across which they had destroyed? Moreover, the Boers had been preparing for some days to give the British a warm reception at this particular point, and naturally did their best to hold their town.

The second of the speeches made by Mr. Chamberlain last week has caused a tremendous sensation in all parts of the world. Some people declare that this is exactly what the Colonial Secretary anticipated and desired. He is certainly not the kind of man to mind a scolding, whether it be given him by Americans, Germans, French, or Britons. He should possess better information than outsiders; but while several organs of the American press aver that the alliance he spoke of is impossible, many German organs have administered douches of cold water on the idea. Frenchmen are naturally angry at being made the object of a warning which looked like a threat, and it cannot be said that the revelation which was the feature of the speech has, on the whole, been very favourably received in England. I gather the predominant feeling here to be this—that, though Mr. Chamberlain is a splendid fighter, and has brilliantly justified the war in South Africa, he is not quite in his element when he ventures on the unfamiliar paths of diplomacy. With all that he said in favour of a good understanding between this country and America and Germany there is great sympathy; only why not call it an understanding merely? To be sure, Mr. Chamberlain corrected himself, but the corrections of statesmen of prominence in certain circumstances are sometimes regarded as confirmation of the original statement. Nor is it in the least degree likely that he wishes in any way to promote bad feeling between England and France; but if he had been trained in the school of Lord Salisbury, or Lord Rosebery, he would probably either have remained silent about the gutter prints of Paris, or couched his rebuke in such a manner that it would have been warmly endorsed by all the public men on the other side of the Channel whose good opinion is worth having.

You will learn with much sorrow of the death of Sir Henry Tate. He not only gave the splendid picture gallery which bears his name to the nation, founded five libraries in South London, and contributed a large sum to University College, Liverpool, but the Royal Infirmary, the Hahnemann Hospital, and the Training Home for Nurses in Liverpool were enriched by his benefactions. The vast wealth which he made, chiefly by the sale of "cube sugar," was employed with no niggard hand for the benefit of his less fortunate fellow-creatures.

### A Book and its Story.

### A NOVEL BY A DUCHESS.

The fact of a book being written by a Duchess is, to a certain class of readers, an attraction in itself, apart from any literary claims it may have. In "One Hour and the Next"* the fair writer, who, as Duchess of Sutherland, is one of the most charming of a bevy of sisters notably gifted with abilities above the average and good looks in proportion, proves that her claim to intelligence of a high order is neither unfounded nor unsustained. Some ten years ago she brought out a book of "impressions" made from notes collected when travelling, and though short contributions from time to time have appeared from her pen, we believe that this is her first attempt at a "real story." And very real it is; and on the last subject one would have imagined the authoress to have chosen, viz., Socialism. The "masses" find infinite interest in reading of the so-called real, but generally fictitious proceedings, of the "classes." To a duchess has been reserved the distinction of writing on a movement emanating directly from the "classes" themselves.

The interest of the book centres in four people, three men and a girl. The girl is Agnes Stanier, and the three men are severally Philip Stanier, her father; Philip Assheton, her lover; and Robert Lester, the hero, a very real creation, if a very unheroic creature. The three men have a strange likeness to each other in a certain strong persistency in the ordering of their lives, except in Philip Stanier's case, in which persistency is but a cloak for a weakness of character, passing otherwise as strength. It was into a shadowed home circle that the heroine, Agnes Stanier, was born. Her father came of an old Midland family possessing a rather unusual capacity for studiousness and culture, a characteristic more strongly developed than mental strength. Personally Philip Stanier had no worldly ambitions, and he took the money lavishly provided by his father almost unconsciously. A student by nature, his one absorbing passion was for knowledge itself. Before he had entered at Oxford he received a severe shock in the sudden death of his father who, in consequence of incautious investments and the dishonesty of the family lawyer, left his wife and son almost penniless. A 'Varsity career being now impossible, he retired to a house in Stoneyard, the manufacturing town where the scene of the story is laid, and opened a school "for young gentlemen whose fathers paid handsomely to give them the classical education which their dull brains could scarcely assimilate." "Patiently the schoolmaster toiled through the years, the wreck of his life ever before his eyes, and the while from his iron self-control a certain weakness grew, like a parasite, on his best self. Adapted as his existence was to the trend of circumstances, he presented ever an obstinate bearing towards the spirit of the new teaching. He shrank from the startling notions and paradoxes of the modern school of thought. With his vision trained to one point of view, in the earlier years of his suffering he dared not, in the later he could not, look to the right or the left." At eight-and-twenty he married a girl "of position and wit," but devoid of the inseeing sympathy necessary to probe the crust of reserve which hid the tender heart underlying it. "She died three years later, crying to him, as he stood dry-eyed in his misery, that he had killed her." Their two children, of whom Agnes was one, were taken in charge by an indignant aunt. It was not until she was in her eighteenth year, and fresh from a high school education, that Agnes again appeared on the scene. "She put her arms round her father's neck with pretty protests, and sat at his feet in the garden during the long August evenings, chattering from the fulness of her fancy. He listened, love in his eyes, but a sinking at his heart." At the moment of her return, Stoneyard was agitated by threats of a strike. This to Agnes had all the elements of excitement failing any other. Of the sufferings of the mill-hands she had no knowledge; the whirr of the mill machinery had always an attraction for her, "but the sight of white-faced women and girls streaming out from their work caused her to turn swiftly away as from infected creatures." One afternoon she had wandered out, oppressed by the hopeless dulness of her life, and longing for something into which her young energies could be thrown, when across her vision came the tall, striking form of Lester, a Socialist agent nominally, but in reality a man of property, with a restless, egotistical temperament and a tragic story in his life; a man to whom real suffering in any form was so repulsive that to escape from its presence he had left an afflicted wife whom he loved, to throw himself into the contest raging between master and man, in order to escape from the demon of dissatisfied self, hoping thus to elude its withering influence. To Agnes Stanier he comes as the Hero, sacrificing himself and his interests to the general good. His advertisement for a typewriter and secretary suggests that here was an opportunity for allying herself to a cause and finding an outlet for unused energies. She joins Lester as his secretary. There is, of course, protest from her father, and from Philip Assheton, his classical master, who loves Agnes. He is also a Socialist; but his Socialism takes the form of Reformation, not Revolution, in the character of the individual first, and in his circumstances later. This Lester neither believes, nor realises. His creed is one of Destruction—Annihilation—the undermining of all existing institutions, sacred and secular. Here is an extract from a discussion between the two men: "In Philip Assheton's voice rang the tone of earnest sincerity, of inspiring faith in the best, that was the keynote of his character. . . . He believed in the good in mankind as much as Lester disbelieved in it; he wanted to work through it, and Lester against it."

"Education, discipline, life, what do you want with them?" said Lester. "To make men reason, to see for themselves that there is nothing worth having anywhere? that all is rotten, rotten, rotten?"

"It is mere lunacy to flaunt your suicidal theories," replied Assheton, "in the face of the working population. You may be able to steer them through without shipwreck, but you are dragging these men from one state to another that is certainly worse than the first."

"Oh," replied Lester, drinking off his whisky and soda, "it throws a little excitement into their existence and relieves its monotony."

When from the first Agnes Stanier comes under the glamour of Lester's personality to the last moment of slow, agonising disillusionment, her whole existence is dominated by his influence; an influence not consciously exercised, nor taken advantage of by him when discovered—for with all his failings Lester was a gentleman, if a very selfish one. He worked for the cause and for a certain measure of popularity. She worked for the cause and for him, and love made the drudgery light; by so doing was she not also identifying herself with the beloved? It is not till the very end of the book that a glimpse is given of Lester and his beautiful tortured wife. This is one of the best chapters in the book, thrilling with intensity. The interest of the story never wavers, but gathers force as it grows, until the final scene is reached, in the awakening of Agnes to the unreality of the vision in which Lester had moved as her Hero and idol. He had never loved her, and in the cruel moment of her disillusionment she turns at last to her father, until then, scorned and repulsed. "Agnes," he said hoarsely. She staggered a few steps towards him. "Father," she cried, "father!" He gathered up the fainting form strongly. He held her with surpassing tenderness, his head against her curls. "Come home, my poor little girl," he said. "Come home."

* "One Hour and the Next." By Millicent Sutherland. (Methuen and Co. London. 6s.)

### Everybody's Opinion.

[Correspondence on all subjects is invited, but we cannot in any way be responsible for the opinions expressed by our correspondents. No communication can be entertained if the name and address of the correspondent is not given, as a guarantee of good faith but not necessarily for publication, or unless one side of the paper only is written on.]

### UNTRAINED NURSES.

"C. R." writes: I am glad to see that the question of untrained nurses is coming to the front again. Can nothing be done to stop this infamous cheating? I know personally two women, one with three months', the other with six weeks' maternity training. They are now working for a small nursing institution, doing medical and surgical work, taking £2 2s. per week. The other day, on going to a case, the doctor asked me if I could take a patient's temperature. I replied, "Yes, certainly; I am a trained nurse." "Oh," he said, "that's nothing, all the nurses have certificates now, and few know a clinical thermometer." Of course we are all judged alike. Because these unscrupulous women know nothing, I and many others in like position are supposed to know nothing too, and though I hold certificates for three years' general work, massage, midwifery, and the L.O.S. certificate, I receive the same fee as they, and am asked if I can take a temperature! Yet we are powerless to help ourselves. It will be a grand day for nurses when these impostors are made to take their proper place.

### LARGE AND SMALL HOSPITALS.

"Lily" writes: "Thistle" also is able to make "sweeping statements," and I think in future, before she attempts to criticise a letter, she ought to at least read it properly. I made no sweeping assertion about nurses trained in large hospitals, but I read a few weeks ago an article in which small hospitals in general were spoken of in such an insulting manner that I could not resist a defence. I don't approve of paper quarrels in the least, but I just want to inform "Thistle" that I spoke of one large fever hospital—not all; and I should be very sorry to think that there were many such in the world. I mentioned this one as a proof that all large hospitals are not infallible any more than small ones, and that good or better work can be done in a small one—of course on a smaller scale. "Thistle" tried to be very sarcastic and very cutting in her remarks, which are really beneath my notice; but I must thank her for the good laugh I enjoyed on reading her communication. I could not help saying aloud to myself, "Well, if the cap fits, let her put it on by all means, though it was not intended for her." As to examinations, a very inferior nurse might pass highest; it is very much a matter of memory. I have known probationers who have had to give up nursing on account of not being able to pass, and yet be real good, kind, practical nurses; while, on the other hand, sometimes those who pass with flying colours are practically worthless nurses. "Thistle" will be kind enough to notice that I have said "some," and that this is not a sweeping assertion any more than the others. Indeed, she has taken my letter up in the most absurd way altogether; she didn't understand in the least that I do not belong to the hospital with the patient with the bed-sores, nor that I was shocked at such an occurrence under any circumstances.

### COTTAGE HOSPITAL MANAGEMENT.

"A Matron of Another Cottage Hospital" writes: I was very interested in an "Honorary Member's" account of the unreasonableness of some members of hospital committees. Again I see that committees—at any rate those of small hospitals in small towns—are of far more nuisance than they are of use. These committees are usually made up of people who understand nothing of hospital management or nursing, but who of course imagine that they know far more than the trained nurse in charge. Surely it is much better for both nurse and patients to allow the nurse full control, provided the right choice is made, for she must be a sensible and conscientious woman as well as a good nurse. It is quite against a nurse's interest to neglect the interests of her patients, and no good nurse would think of neglecting a bad case in order to go out; but it is for her, the trained nurse, to decide what is neglect, not the committee. Amateur nursing generally consists of a great deal of apparent fussiness and very little real nursing; professional nursing of no unnecessary fussiness, but of real and necessary nursing. The professional nurse knows when she can best leave her patient. It is as much a duty to the patient as it is to herself to take some relaxation, and especially should she have as much outdoor exercise as possible. How can one keep bright and be patient if one has no change? I should like the committee of that Memorial Hospital, referred to in one of your recent issues, to understand that a bright, happy, patient nurse is a great factor in the recovery of her patients. Nursing does not consist in giving medicines, making poultices, and shaking pillows. No, it is far more. In some subtle way, partly because of the very susceptibility of a sick person in their helplessness, we can give out healing in our very touch, in our very manner of speaking and acting—thus we need to keep both mind and body in good tone. I have charge of a cottage hospital in a small northern town, containing six beds and one cot. My average number of cases for the year, and amount and nature of work, correspond very much to those of the previous-named hospital, but—and this just makes the difference—I have not an unappreciative, fault-finding committee to contend with. If I had, I could not do my work as well. At present, everything goes smoothly and pleasantly, and the expenses would compare favourably as to economy with any other similar institution. The cases have always been successful and recovered, where success and recovery were possible. I have a very happy life, and, taking one month with another, not at all hard work. Of course, there are times when I am unable to leave, but then there also times when I am out bicycling for hours, with no fear of the unfriendly censure of a committee. I am thankful to add that I have not to ask the permission of the committee to buy every pennyworth of pins or yard of tape I may require for the hospital, but that these matters are left to my own discretion; in other words, that I am trusted, which, after all, is the greatest incentive to good work and management.

### Winter Sale of Ladies' Work.

The Working Ladies' Guild have been fortunate in securing the loan of Grosvenor House for their winter's sale of work, which is being held this week. Princess Henry of Battenberg opened it on Wednesday, and presided at one of the stalls. She was received by the committee, and inspected the beautiful work on every stand before taking her place at her own. Her Royal Highness was accompanied by Miss Bulteel. Lady Constance Shaw Lefevre, Lady Eden, Lady Cunningham, and Mrs. Macmillan presided over the stalls of the various departments of ladies' work, namely, art needlework, decorative art, and plain needlework. Excepting some charming pottery and foreign trinkets, everything was the handiwork of poor ladies, protected, encouraged, and sometimes taught by the Guild. The standard of excellence is very high, and the same thorough workmanship was observable in the clothing for the poor—of which there was a large store—as in the dainty children's garments, the embroidery, marqueterie lace, and French pointing. Much attention was given to the repairs in process of execution on a large piece of valuable old tapestry, the skilful needles of the ladies engaged restoring it to first excellence.

### Death in Our Ranks.

We regret to record the death, on December 1st, of Sister Louisa Langton, of the Infirmary, St. George's-in-the-East, from typhoid fever. Sister Langton was a kind and conscientious nurse, and her death is keenly felt by a large circle of nurses. The interment took place on Tuesday.

### For Reading to the Sick.

"And of His fulness have we all received, and grace for grace."—*S. John* i. 16.

O God, O kinsman, loved, but not enough!
O Man, with eyes majestic after death,
Whose feet have toiled along our pathway rough,
Whose lips drawn human breath;
By that one likeness which is ours and Thine,
By that one nature which doth hold us kin,
By that high heaven where sinless, Thou dust shine
To draw us sinners in;
Come! lest this heart should, cold and cast away,
Die ere the guest adored she entertain—
Lest eyes which never saw Thy earthly day
Should miss Thy heavenly reign! —*Jean Ingelow.*

**Reading.**

The Incarnation and Passion of the Son of God is the breaking of the power of sin. By the Incarnation there appeared One who lived without fault—One over whom, from the moment of His conception to that of His death, sin had no dominion. Just as God in Christ is revealed as interposing to remedy man's inherited defect of birth sin, so also is He manifested as the liberator of man from the chain and habit of actual sin.—*Staley.*

Christian holiness is the reproduction in the individual of the life of the Incarnate Son of God. That this might be possible, there took place that series of events which St. John describes as the glorification of Jesus Christ. The life, perfectly well-pleasing to God, and therefore the supreme standard of holiness, passes through the stage of death. The sacrifice on Calvary removes the barrier raised between the Creator and His creatures by sin. The resurrection is, on the one hand, the seal of God's acceptance stamped upon His Son's atoning work; on the other, marks the final stage in that process by which Christ's human nature is "perfected." For by the resurrection that nature is spiritualised, is released from earthly limitations, and becomes available as a recreative force. The ascension is the condition of Christ's manifestation as a "quickening Spirit," as the "power of God." By sacramental channels he communicates to our entire nature His life-giving humanity, as the means of our recreation after the image of God. Thus the life of the Incarnate is extended in the life of the redeemed, and by a natural and orderly growth the character of Christ is reproduced in His members through the continuous operation of the Spirit, whose office it is to "take of the things of Christ and show them unto" men. He who is outwardly our example thus becomes an inward principle of life.—*R. L. Ottley.*

Holy Saviour, Friend unseen,
Since on Thine Arm Thou bid'st me lean,
Help me throughout life's varying scene,
By faith to cling to Thee!

Blest with this fellowship divine,
Take what Thou wilt, I'll ne'er repine.
E'en as the branches to the vine,
My soul would cling to Thee!

Far from her home, fatigued, opprest,
Here she has found her place of rest;
An exile still, yet not unblest
While she can cling to Thee!

Without a murmur I dismiss
My former dreams of earthly bliss;
My joy, my consolation this,
Each hour to cling to Thee!
—*C. Elliott.*

### Notes and Queries.

The contents of the Editor's Letter-box have now reached such unwieldy proportions that it has become necessary to establish a hard and fast rule regarding Answers to Correspondents. In future, all questions requiring replies will continue to be answered in this column without any fee. If an answer is required by letter, a fee of half-a-crown must be enclosed with the note containing the enquiry. We are always pleased to help our numerous correspondents to the fullest extent, and we can trust them to sympathise in the overwhelming amount of writing which makes the new rules a necessity.

Every communication must be accompanied by the writer's name and address, otherwise it will receive no attention.

*Circumcision.*

(108) Will you kindly tell me the necessary things to be ready for the doctor's use for a circumcision?—*A. E. H.*

The presumption is that the surgeon will bring his instruments. The nurse should take care that the parts concerned have been very thoroughly washed with soap and water; she should provide plenty of boiled water; and it would be well to have at hand a couple of thoroughly cleaned sponges, although most surgeons now use artificial sponges which they bring with them.

*The Infection of Puerperal Fever.*

(109) Will you kindly say if puerperal fever can be conveyed to a female dog which was caressed by the nurse who was attending the case mentioned recently in the "Nursing Mirror"?—*Anxious.*

There seems no likelihood of the occurrence of the catastrophe suggested.

*Materia Medica.—Corpse.*

(110) Could you tell me the name of the author and address of the publisher of a "Materia Medica" that was mentioned in The Hospital a few months back? 2. I should be glad if you could tell me which is the best way to keep a corpse in good condition? Is it better to ventilate the room or exclude all fresh air? I am living in a country district, and they do not believe in ventilation at any time.—*District Nurse.*

The Scientific Press, London, W., would always procure and forward books on nursing and medical subjects. The "Materia Medica" to which you refer was probably Mitchell's. 2. Certainly ventilate the room, but be careful to keep the flies out. Use plenty of disinfectants, also ice if it can be procured.

*Climate and Outfit.*

(111) 1. What sort of climate is there in the Transvaal? 2. What kind of [illegible]erclothes and outfit generally would a nurse require if called out by [illegible]e War Office for service in the Transvaal?—*B. M.*

The climate is usually hot and dry, alternating during the rainy season with tremendous rains and thunderstorms, with cold nights at certain seasons of the year. 2. The same underwear as for a hot English summer, plenty of thin flannels, muslin, and print dresses, with a warm coat and skirt and dressing gown. The nurses sent out by Government will of course be provided with uniform, which have lately been fully described in the "Mirror."

*Red Cross Nurse.*

(112) Will you kindly let me know what constitutes a "Red Cross Nurse," and if there is any special training necessary for same; also if only "Red Cross Nurses" can join the Army Reserve Service?—*One who is Anxious to Know.*

Any nurse with three years' training and general experience can be, if otherwise suitable, employed on the Army Nursing Reserve. When nurses are engaged nursing the sick and wounded in war under the conditions laid down by the Convention of Geneva, they wear a red cross. This badge ensures them protection from both combatants.

*Plague Duty.*

(113) It was stated in the "Mirror" of a few weeks ago that nurses on plague duty in India are obliged to board themselves. Will you kindly tell me if this is correct, as I have always been under the impression that free quarters, board, &c., are allowed by the Government?—*Nurse B.*

All depends upon the locality in which a nurse is employed. The paragraph in the "Mirror" was from a private source, and fully authenticated.

*Address.*

(114) Can you kindly give me the address of the Women's Employment Bureau, opened some time ago to provide employment for gentlewomen of small means?—*An Inquirer.*

No. 60, Chancery Lane.

*Dispensing.*

(115) My daughter (aged 18) is desirous of going in for dispensing, and our medical man has referred me to you for information as to how she is best to proceed. Will you kindly favour me with the necessary directions?—*W. L. G.*

You must write to the secretaries of the Pharmaceutical Society, 17, Soho Square, Bloomsbury; and of the Apothecaries' Hall, Blackfriars, and ask for the information you require. If your daughter ever wishes to act independently she must have the certificates of the latter, as the former only qualifies her as assistant dispenser. See "Mirror" for July 1st, "Dispensing up to Date."

(116) Will you kindly tell me the names of books which would be best to study to enable me to teach myself dispensing? I am very anxious to qualify as a dispenser, but at present I have not the opportunity of attending classes. Will you also please tell me whether the fee for attending classes would amount to very much, as, if so, I could not afford it? I do know enough of dispensing to make up a doctor's prescription, and have done so on several occasions. Before ending my letter I must tell you how very much I appreciate The Hospital, and how many times I have been helped by it in many ways.—*Nurse.*

Dispensing cannot be learned from books. See answer to "W. L. G

